# Fully Closed Genome Sequences of Five Type Strains of the Genus *Cronobacter* and One *Cronobacter sakazakii* Strain

**DOI:** 10.1128/genomeA.00142-16

**Published:** 2016-03-24

**Authors:** Deborah Moine, Mohamed Kassam, Leen Baert, Yanjie Tang, Caroline Barretto, Catherine Ngom Bru, Adrianne Klijn, Patrick Descombes

**Affiliations:** a Nestlé Institute of Health Sciences SA, EPFL Innovation Park, Lausanne, Switzerland; b Nestec Ltd., Nestle Research Center, Lausanne, Switzerland

## Abstract

*Cronobacter* is associated with infant infections and the consumption of reconstituted infant formula. Here we sequenced and closed six genomes of *C. condimenti*^T^, *C. muytjensii*^T^, *C. universalis*^T^, *C. malonaticus*^T^, *C. dublinensis*^T^, and *C. sakazakii* that can be used as reference genomes in single nucleotide polymorphism (SNP)-based next-generation sequencing (NGS) analysis for source tracking investigations.

## GENOME ANNOUNCEMENT

*Cronobacter* (formerly *Enterobacter sakazakii*) is a foodborne pathogen that has been identified as the causative agent of severe clinical complications in neonates and infants, such as meningitis, necrotizing enterocolitis, and septicemia (1, 2). The origin of this pathogen is not clear, but *Cronobacter* has been isolated from a wide range of foods, among which powdered infant formula (PIF) has been identified as the dominant vehicle of transmission (3, 4). *Cronobacter* is also often isolated from the environment and can be found in soil samples, domestic kitchens, and predominantly PIF manufacturing facilities (5, 6). The genus *Cronobacter* represents *E. sakazakii*, which was reclassified in 2007 as a result of biotyping and genotyping studies (7, 8).

Reliable identification and discrimination of *Cronobacter* strains is of importance due to the severe illness and ubiquitous occurrence in the environment and food. A multilocus sequence typing (MLST) scheme (9) has been shown to enable differentiation of closely related *Cronobacter* strains. The high discriminatory power and the drop in the cost of the next-generation sequencing (NGS) technologies favor the use of NGS as a routine diagnostic tool in public health reference laboratories in the near future (10). Clustering of *Cronobacter* isolates based on NGS data will allow a powerful source-tracking analysis. The clustering and the creation of phylogenic trees based on single nucleotide polymorphism (SNP) analysis of the NGS data are carried out by mapping short read sequences of *Cronobacter* isolates to a reference genome. The identification of reference genomes is essential for a reliable SNP-based analysis. Only a few complete genomes of *Cronobater* are available in public databases and the pool of reference genomes needs to be extended. Therefore, an effort was done to sequence and close genomes of *Cronobacter* spp. that can be used in SNP-based NGS analysis to support detailed source tracking investigations.

Genomic DNA was extracted from midexponential cultures using a Gentra DNA Purgene kit (Qiagen), and then 20-kb libraries were prepared following Pacific Biosciences (PacBio) protocol and Blupippin size selection. Sequencing was performed on the PacBio RSII platform using P4/C2 chemistry (P6/C4 for *C. malonaticus*) and three to four single-molecule real-time (SMRT) cells were used per strain with a 180-min (240 min for *C. malonaticus*) collection protocol. The subreads were *de novo* assembled using the PacBio Hierarchical Genome Assembly Process (HGAP)/Quiver software package (11), followed by minimus2 for genome circularization (12) and final polishing with Quiver. All the strains were assembled into a single contig corresponding to the chromosome. For some strains one to four circular plasmids were also obtained. The nucleotide sequences have been deposited at NCBI. The results of the sequencing and assemblies are summarized in Table 1. The genomes were annotated using the NCBI Prokaryotic Genomes Automatic Annotation Pipeline (PGAAP) and have been deposited at GenBank (NCBI).

**TABLE 1:** Summary of genome sequencing and nucleotide accession numbers

Organism	Chromosome size (bp)	No. of plasmids	Plasmid size (bp)	Accession no.
*C. condimenti* LMG 26250, CECT 7863^T^	4,366,820	1	164,790	CP012264 to CP012265
*C. muytjensii* ATCC 51329^T^	4,385,738	0	NA^*a*^	CP012268
*C. sakazakii* NCTC 8155	4,348,995	3	124,048/117,750/53,771	CP012253 to CP012256
*C. universalis* NCTC 9529^T^	4,323,715	1	136,454	CP012257 to CP012258
*C. malonaticus* LMG 23826, DSMZ 18702^T^	4,294,640	2	126,501/52,758	CP013940 to CP013942
*C. dublinensis* LMG 23823, DSMZ 18705^T^	4,444,709	1	203,534	CP012266 to CP012267

a NA, not applicable.

During sequencing, epigenetic modifications of each nucleotide position were measured as kinetic variations (KVs) in nucleotide incorporation rates. Motifs were deduced from the KV data (13). Analysis were done using SMRT portal RS_Modification_and_Motif_Analysis Protocol.

### Nucleotide sequence accession numbers.

Sequences have been deposited in GenBank under the accession numbers listed in Table 1. Raw reads and motif summaries are deposited at SRA: *C. condimenti*^T^ SRR2154341, *C. muytjensii*^T^ SRR2154340, *C. sakazakii* SRR2154342, *C. universalis*^T^ SRR2154343, *C. malonaticus*^T^ SRR3112550, and *C. dublinensis*^T^ SRR2154345.
